# Body Mass Index and 10-Year Clinical Outcomes After Percutaneous Coronary Intervention—Interaction with Age, Sex, Diabetic Status and Clinical Presentation

**DOI:** 10.3390/jcm14051413

**Published:** 2025-02-20

**Authors:** Gjin Ndrepepa, Sebastian Kufner, Salvatore Cassese, Michael Joner, Erion Xhepa, Hendrik B. Sager, Heribert Schunkert, Adnan Kastrati

**Affiliations:** 1 Department of Cardiology, Deutsches Herzzentrum München, TUM Universitätsklinikum, Lazarettstrasse 36, 80636 Munich, Germany; sebastian.kufner@tum.de (S.K.); cassese@dhm.mhn.de (S.C.); joner@dhm.mhn.de (M.J.); xhepa@dhm.mhn.de (E.X.); hendrik.sager@tum.de (H.B.S.); schunkert@dhm.mhn.de (H.S.); kastrati@dhm.mhn.de (A.K.); 2 German Center for Cardiovascular Research (DZHK), Partner Site Munich Heart Alliance, Munich, Germany

**Keywords:** body mass index, coronary artery disease, mortality, obesity, percutaneous coronary intervention

## Abstract

**Background/Objective**: The association of body mass index (BMI) with long-term outcomes following percutaneous coronary intervention (PCI) remains poorly investigated. We undertook this study to assess the association between BMI and long-term outcomes after PCI. **Methods**: Overall, 5597 patients with coronary artery disease undergoing PCI were included in the study. Patients were categorized in groups according to the following BMI categories: underweight group (BMI <18.5 kg/m^2^), normal weight group (BMI 18.5 kg/m^2^ to <25 kg/m^2^), overweight group (BMI 25 kg/m^2^ to <30 kg/m^2^) and obesity group (BMI ≥30 kg/m^2^). The primary endpoint was all-cause mortality at 10 years. **Results:** At 10 years, all-cause deaths (primary endpoint) occurred in 1754 patients: 31 deaths (59.7%) in the underweight group, 582 deaths (39.1%) in the normal weight group, 710 deaths (31.1%) in the overweight group and 431 deaths (33.8%) in the obesity group (overall *p* < 0.001; *p* for nonlinearity <0.001). Nonsurvivors had a significantly lower BMI compared with survivors (26.5 [24.2–29.9] kg/m^2^ vs. 27.2 [24.8–30.1] kg/m^2^, *p* < 0.001). Interaction testing showed a BMI-by-age interaction denoting a stronger association between higher BMI (≥25 kg/m^2^) and reduced risk of all-cause mortality in patients ≥75 years of age (P_int_ = 0.009). The association of BMI with all-cause mortality was U-shaped (*p* for nonlinearity < 0.001). The C-statistic of the multivariable Cox proportional hazards model for mortality increased from 0.762 [0.751–0.773] with baseline variables only to 0.766 [0.756–0.777], *p* < 0.001) after the BMI inclusion in the model (baseline variables plus BMI). **Conclusions**: In patients with coronary artery disease undergoing PCI, BMI was associated with 10-year mortality with a U-shaped relationship.

## 1. Introduction

The prevalence of obesity has reached epidemic proportions with approximately 38% of the world population having a body mass index (BMI) of >25 kg/m^2^ and the projections suggest a further increase in the near future [1]. The Global Burden of Disease (GBD) 2015 Obesity Collaborators estimated that a high BMI accounted for 4 million deaths in 2015 with nearly 70% of them being related to cardiovascular disease [2]. Patients with obesity develop coronary artery disease (CAD) at a younger age [3] and have a shorter life span compared with subjects with a normal weight [4]. A meta-analysis with >300,000 patients and 18,000 registered acute coronary events showed that BMI in the overweight and obesity categories was associated with an increased risk for coronary events [5]. Obesity promotes CAD via a number of mechanisms including insulin resistance, endothelial dysfunction, sympathetic nervous system activation, atherogenic profile of plasma lipids, increased vascular resistance, increased inflammatory burden and prothrombotic state [6,7,8]. Despite this evidence, obesity has not been consistently found to be a risk factor for cardiovascular disease outcomes. In particular, an increased risk of in-hospital complications and one-year mortality after percutaneous coronary intervention (PCI) in patients with a BMI < 18.5 kg/m^2^ has been reported and the term “obesity paradox” has been coined by Gruberg et al. [9] to describe an inverse association between BMI and mortality after PCI. In the past, the association between BMI and outcome after PCI has been extensively investigated, yet a number of issues need further clarification. First, the association between BMI and mortality after PCI was mostly investigated in short-term studies [7] and the inverse association between BMI and mortality after coronary revascularization appears to wane at long-term follow-up particularly when severe obesity is considered [10,11]. Second, the BMI–mortality association has been most commonly studied and the association of BMI with other outcomes after PCI remains poorly investigated. Third, the BMI–mortality association has been investigated in BMI categories defined according to the World Health Organization (WHO) recommended cut-offs. However, the best cut-offs that define BMI categories associated with lower or higher mortality are unknown. Fourth, although each BMI category may have a distinct pattern of association with mortality in terms of strength and direction, not rarely, the BMI–mortality association was investigated in combined BMI categories (i.e., underweight/normal weight or overweight/obesity categories grouped together). Fifth, it is not known whether there are differences in the association of BMI with mortality due to cardiac or noncardiac causes. Against this background, we conducted this study with the following aims: first to investigate the association of BMI with the outcomes after PCI over a 10-year follow-up; second to assess the association of BMI with mortality following PCI across the entire spectrum of BMI and define the BMI cut-offs (and segments) associated with the lowest or highest risk of mortality; third, to investigate whether there are differences in the association between BMI and cardiac or noncardiac mortality and whether the BMI–mortality association differs in groups of patients categorized according to age, sex, diabetic status and clinical presentation.

## 2. Methods

### 2.1. Patients

This study comprised 5597 patients who underwent PCI with stent implantation from September 2007 until August 2009 in two university hospitals in Munich, Germany. Patients were obtained from 2 randomized controlled trials: the Intracoronary Stenting and Angiographic Results: Test Efficacy of 3 Limus-Eluting Stents (ISAR-TEST) 4 randomized study (NCT00598676; 2603 patients) [12], and the Intracoronary Stenting and Angiographic Results: Test Efficacy of Sirolimus- and Probucol- and Zotarolimus-Eluting Stents (ISAR-TEST) 5 randomized study (NCT00598533; 3002 patients) [13]. Patients were 18 years or older, had ischemic symptoms or evidence of myocardial ischemia (spontaneous or inducible) and coronary artery stenoses with ≥50% lumen obstruction documented in coronary angiography. Patients with target lesion located in the left main coronary artery, cardiogenic shock, cancer (or other diseases) with a life expectancy of <12 months, allergy to the study drugs or pregnancy were excluded. Of the 5605 patients recruited in the source trials, 5597 patients had BMI data available and these patients were included in the current analysis. Written informed consent and institutional ethics committee approval were obtained at the time of the patient’s recruitment in the source trials [12,13]. The study was conducted in accordance with the Declaration of Helsinki.

### 2.2. Study Definitions and Measurements

BMI was calculated using the patient’s height and weight measured at the time of patient’s recruitment in the source trials. The diagnosis of CAD required angiographic documentation of stenoses with ≥50% lumen obstruction in the native coronary arteries. Arterial hypertension, dyslipidemia, type 2 diabetes and smoking were defined according to the guideline-recommended criteria at the time of patient’s inclusion in the source studies. Left ventricular ejection fraction was measured by angiography using the area-length method. The estimated glomerular filtration rate (GFR) was calculated using the Chronic Kidney Disease Epidemiology Collaboration (CKD-EPI) equation [14]. Serum creatinine was measured using the compensated Jaffe method. Patients were categorized in groups according to the BMI cut-offs: underweight group (BMI < 18.5 kg/m^2^), normal weight group (BMI 18.5 kg/m^2^ to <25 kg/m^2^), overweight group (BMI 25 kg/m^2^ to <30 kg/m^2^) and obesity group (BMI ≥ 30 kg/m^2^).

### 2.3. Outcomes and Follow-Up

The primary endpoint was the incidence of 10-year all-cause mortality. The 10-year incidences of cardiac mortality and noncardiac mortality, nonfatal myocardial infarction, definite stent thrombosis, target lesion revascularization (TLR), target vessel revascularization (TVR) and nontarget vessel revascularization (NonTVR) were also assessed. Cardiac death and definite stent thrombosis were defined according to the 2007 Academic Research Consortium (ARC) criteria [15]. All other deaths were defined as noncardiac deaths. Myocardial infarction was diagnosed using the 2007 Universal Definition of Myocardial Infarction criteria [16]. TLR was defined as a repeat stenting or balloon angioplasty of the stented lesion, including 5 mm borders adjacent to the stent. TVR was defined as a repeat coronary intervention (PCI or coronary artery bypass surgery) in any segment of the target vessel, including the target lesion. NonTVR was defined as a repeat revascularization of a coronary vessel other than the target vessel.

The follow-up included telephone calls or office visits at 1 and 12 months and annually up to 10 years. All outcomes were assessed and adjudicated by the event adjudication committee, in the setting of the source trials.

### 2.4. Statistical Analysis

Continuous data with skewed distribution are presented as the median with 25th–75th percentiles and compared with the Kruskal–Wallis rank sum test. The distribution pattern of continuous variables was tested with the Kolmogorov–Smirnov test. Categorical data are presented as counts and proportions and compared with the chi-squared test. The primary endpoint (all-cause deaths at 10 years) was presented as cumulative incidence according to the Kaplan–Meier method. The other outcomes are shown as cumulative incidences after accounting for competing risk of death. Differences between the groups according to the BMI cut-offs were assessed using the univariable Cox proportional hazards model. The association of BMI with 10-year outcomes was adjusted in the multivariable Cox proportional hazards model. The following variables were entered into the model: BMI (as a continuous or category variable), patient’s age, gender, type 2 diabetes, current smoking, history of arterial hypertension, history of hypercholesterolemia, prior myocardial infarction, prior coronary artery bypass graft surgery, clinical presentation (acute coronary syndrome or chronic coronary disease), extent of angiographic CAD, estimated GFR, target coronary vessel, lesion complexity and left ventricular ejection fraction. BMI was entered into the multivariable model(s) as a continuous or category variable (in different Cox proportional hazard models). Incomplete baseline data were imputed using the predictive mean matching. The interaction testing was used to assess whether there are differences in the association of BMI (dichotomized at 25 kg/m^2^) with outcomes in subgroups of patients according to age (<75 years vs. ≥75 years), sex (women vs. men), diabetes mellitus (with vs. without diabetes) and clinical presentation (acute coronary syndrome vs. chronic coronary disease). The restricted cubic spine regression analysis was used to assess a potentially nonlinear association between BMI and 10-year mortality. The BMI was entered as 18.5, 25 and 30 kg/m^2^ knots. The C-statistic of the multivariable Cox proportional hazards models without BMI (with baseline variables) and with inclusion of BMI (baseline variables plus BMI) was calculated to assess whether the discrimination for mortality was improved by the inclusion of BMI into the model. The C-statistics of the mortality models with and without BMI were compared with the CompareC package. The R 4.1.0 Statistical Software (The R Foundation for Statistical Computing, Vienna, Austria) was used for statistical analysis. The statistical significance was defined at a two-sided *p* value of <0.05.

## 3. Results

### 3.1. Baseline Data

This study included 5597 patients: 59 patients (1.1%) in the underweight, 1608 patients (28.7%) in the normal weight, 2509 patients (44.8%) in the overweight and 1421 patients (25.4%) in the obesity groups, respectively. Baseline data are shown in Table 1. History of arterial hypertension, prior myocardial infarction and left ventricular ejection fraction did not differ significantly across the groups. The other variables differed significantly across the groups according to BMI. Procedural data are shown in Table S1. Therapy at hospital discharge is shown in Table S2. The prescription of guideline-recommended drugs at discharge (aspirin, P2Y_12_ inhibitors, statins, angiotensin-converting enzyme inhibitors and beta blockers) did not differ significantly in groups according to the BMI values.

### 3.2. Clinical Outcome

**Figure 1:**
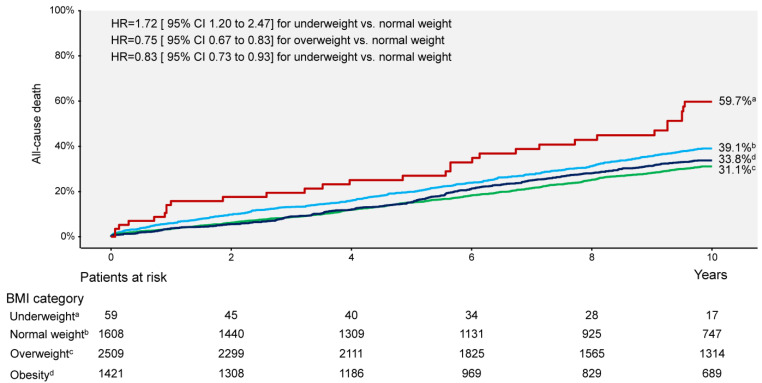
Kaplan–Meier curves of all-cause mortality in patient groups according to categories of body mass index. BMI = body mass index; CI = confidence interval; HR = hazard ratio.

Ten-year clinical outcomes are shown in Table 2. Deaths of any cause (primary endpoint) occurred in 1754 patients (31.3%): 31 deaths (Kaplan–Meier estimate, 59.7%) occurred in patients with underweight, 582 deaths (39.1%) occurred in patients with normal weight, 710 deaths (31.1%) occurred in patients with overweight and 431 deaths (33.8%) occurred in patients with obesity (overall *p* <0.001; Figure 1). Nonsurvivors had a significantly lower BMI compared with survivors (26.5 [24.2–29.9] kg/m^2^ vs. 27.2 [24.8–30.1] kg/m^2^, *p* < 0.001). Cardiac and noncardiac deaths occurred in 1076 patients (61%) and 678 patients (39%), respectively (Table 2 and Figure S1). The rates of myocardial infarction appear not to differ significantly according to the BMI categories. The rates of definite stent thrombosis were higher in patients with overweight and obesity compared with patients with normal weight (reference). The rates of TLR did not differ significantly in patients with underweight, overweight or obesity compared with patients with normal weight, whereas the rates of TVR and NonTVR were higher in patients with overweight and obesity compared with the reference group (Table 2).

Interaction testing showed a BMI-by-age interaction demonstrating a stronger association between a higher BMI (≥25 kg/m^2^) and reduced risk of all-cause mortality in patients ≥75 years of age (P_int_ = 0.009; Figure S2). There were also significant BMI-by-age and BMI-by-sex interactions showing a stronger association between a higher BMI and reduced risk of noncardiac mortality in patients ≥75 years of age (P_int_ =0.013) and women (P_int_ =0.043; Figure S3). There were no BMI-by-age, -sex, -diabetic status or clinical presentation significant interactions regarding cardiac mortality (P_int_ ≥ 0.160), myocardial infarction (P_int_ ≥ 0.192), stent thrombosis (P_int_ ≥ 0.668), TLR (P_int_ ≥ 0.390), TVR (P_int_ ≥ 0.311) and NonTVR (P_int_ ≥ 0.268) for all interactions.

The association of BMI with all-cause mortality was adjusted in the Cox proportional hazards model (the list of characteristics entered into the model is found in the Section 2). When BMI was entered into the model(s) as a continuous variable, the association of BMI with all-cause (hazard ratio [HR] =1.06, 95% confidence interval [CI] 0.97 to 1.16]), cardiac (HR = 0.99 [0.92–1.07]) and noncardiac (HR = 1.10 [0.94–1.27]) mortality was not significant (with all 3 HRs calculated for 5 kg/m^2^ higher BMI). When BMI was entered into the model as a category variable, the risk of all-cause (HR = 1.52 [1.06–2.20]) and noncardiac (HR = 2.47 [1.50–4.05]) was significantly higher in the underweight patients compared with normal weight patients. Overweight patients had a lower risk of all-cause (HR = 0.82 [0.73–0.92]) and noncardiac (HR = 0.82 [0.68–0.99]) mortality compared with normal weight patients. Full results of the multivariable model are shown in Table 3. The association of BMI categories with all clinical outcomes (as compared with normal weight category) is shown in Table 4.

The C statistics of the multivariable models of all-cause, cardiac and noncardiac mortality with baseline characteristics (i.e., without BMI) were 0.762 [0.751–0.773], 0.779 [0.765–0.792] and 0.742 [0.725–0.761], respectively. The inclusion of BMI in the model (i.e., baseline characteristics plus BMI) resulted in a significant increase in the C-statistics of the models of all-cause (0.766 [0.756–0.777], *p* < 0.001), cardiac (0.781 [0.768–0.794], *p* = 0.023) and noncardiac mortality (C statistic: 0.750 [0.732–0.768], *p* = 0.0002), respectively.

**Figure 2:**
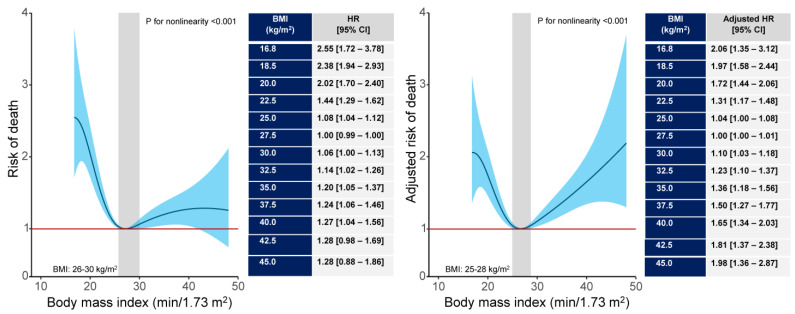
Nonlinear association between body mass index (BMI) with 10-year all-cause mortality in unadjusted (**left panel**) and adjusted (**right panel**) analysis. The spline curves show the risk of mortality across the whole spectrum of BMI values. The shaded areas show the BMI values associated with the lowest 10-year mortality. In unadjusted analysis, the BMI values lower than 26 kg/m^2^ and higher than 30 kg/m^2^ were associated with increased risk of mortality. In adjusted analysis, the BMI values lower than 25 kg/m^2^ and higher than 28 kg/m^2^ were associated with the increased adjusted risk of mortality. The inserted tables on the right side of each graph show hazard ratios (HRs) with 95% confidence interval (CI) for all-cause mortality in various BMI values.

**Figure 3:**
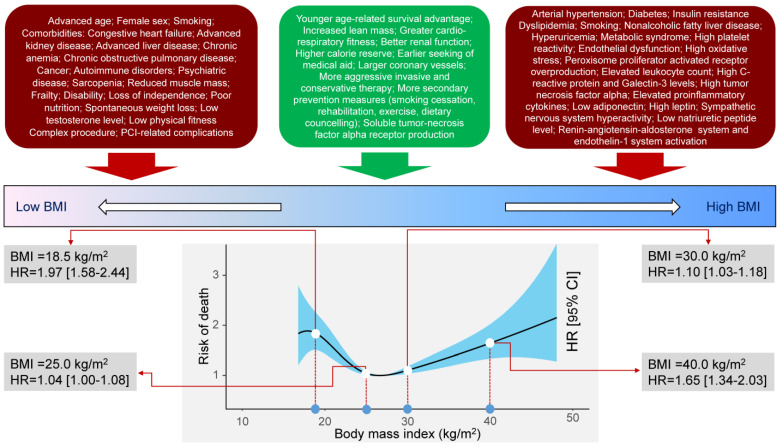
Association between body mass index and all-cause mortality. **Upper part:** Risk factors and comorbidities that tend to cluster in various parts of the body mass index scale. **Lower part:** Restricted cubic spline curve showing the relationship between body mass index and the risk of all-cause mortality. BMI = body mass index; CI = confidence interval; HR = hazard ratio; PCI = percutaneous coronary intervention.

The restricted cubic spline regression analysis showed a nonlinear relationship between BMI and all-cause, cardiac and noncardiac mortality (*p* for nonlinearity <0.001 for all three associations). The association of BMI with all-cause and cardiac mortality was U-shaped. The association of BMI with noncardiac mortality was nonlinear but not U-shaped. BMI values 26 kg/m^2^ to 30 kg/m^2^ and 26 kg/m^2^ to 33 kg/m^2^ were associated with lowest risk of all-cause and cardiac mortality, respectively. For BMI values >25 kg/m^2^, the association between BMI and noncardiac mortality was not significant. In the adjusted analysis, BMI values 25 kg/m^2^ to 28 kg/m^2^ were associated with the lowest risk of all-cause mortality (Figure 2 and Figure 3). BMI values 25 kg/m^2^ to 31 kg/m^2^ and 24 kg/m^2^ to 28 kg/m^2^ were associated with the lowest risk of cardiac and noncardiac mortality, respectively (Figures S4 and S5).

## 4. Discussion

In this study, we investigated the association of BMI with 10-year outcomes after PCI. The key findings of the study can be summarized as follows: (1) BMI was associated with 10-year mortality with a U-shaped relationship. There was a steep increase in mortality with decreasing BMI values within the normal weight and underweight BMI categories and a gradual (less steep) increase in mortality with increasing BMI values in the overweight and obesity categories of the BMI spectrum. (2) BMI values between 25 kg/m^2^ and 28 kg/m^2^ were associated with lowest adjusted 10-year risk of all-cause mortality. For BMI values <25 kg/m^2^ and >28 kg/m^2^, the risk of all-cause mortality was significant. (3) The patterns of association of BMI with mortality appear to differ with respect to cardiac and noncardiac mortality. The association between BMI and cardiac mortality was U-shaped and vulnerable (was attenuated) to adjustment whereas the association between BMI and noncardiac mortality was L-shaped and resistant to adjustment for potential confounders. Patients in the underweight BMI category had a higher adjusted risk of noncardiac mortality whereas patients in the overweight category had a lower risk of noncardiac mortality, both compared with the normal weight BMI category. 4) BMI in the overweight category was associated with a higher risk of definite stent thrombosis and NonTVR, whereas patients in the underweight BMI category had a lower risk of NonTVR than patients in the normal weight BMI category. There was no independent association between BMI and the risk of myocardial infarction, TLR or TVR. 5) There was a BMI-by-age interaction with respect to all-cause and noncardiac mortality suggesting a lower risk of all-cause and noncardiac mortality in patients ≥75 years of age and a BMI > 25 kg/m^2^ and a BMI-by-sex interaction suggesting a lower risk of noncardiac mortality in women with a BMI > 25 kg/m^2^. There were no statistically significant BMI-by-age, -sex, -diabetic status or clinical presentation interactions regarding other outcomes analyzed in the current study.

Many studies support the existence of an obesity paradox in the association between BMI and mortality after PCI [17] even in the current practice of coronary interventions [18,19]. Although the term “obesity paradox” is widely used, it does not apply to the entire association of BMI with mortality. Several studies reported a U-shaped or J-shaped relationship between BMI and mortality after PCI with a higher risk of death on the lower and upper parts of the BMI scale and a lower risk of death in the middle part of the BMI scale corresponding to the overweight BMI category [20]. Other studies did not find a protective effect of obesity with respect to the risk of death after PCI [21,22,23,24]. The association between BMI and mortality after PCI depends on multiple factors including the degree of adjustment in multivariable analyses [25,26,27], age of patients [28], sex [29] and whether the category of severe obesity [30] was considered.

Our study showed a U-shaped association between BMI and 10-year risk of all-cause mortality with the lowest risk of mortality corresponding to the BMI values in the overweight category. Several aspects of this finding may need commenting. First, the association between BMI and 10-year mortality remained U-shaped but the statistical significance was attenuated after the adjustment with BMI entered in the risk models as a continuous variable. This sounds reasonable considering that the risk of mortality increased for BMI values <25 kg/m^2^ and >28 kg/m^2^. Other studies have also shown a nonlinear U-shaped relationship between BMI and mortality with a higher mortality for BMI values <27 kg/m^2^ and > 32 kg/m^2^ [20]. Thus, assessing BMI as a continuous variable and calculating the risk for a given unit over the entire BMI scale may be misleading considering that the association with mortality may differ markedly in direction and strength in the different parts of the BMI scale. The inclusion of BMI in the risk models as a category variable and restricted cubic spline regression managed to delineate a significant association between various categories of the BMI scale and the risk of mortality. Second, our study found that the association of BMI with cardiac and noncardiac mortality differs, both in the pattern and the impact of adjustment on the association. The association between BMI and cardiac mortality was U-shaped. The association between BMI and noncardiac mortality was L-shaped with a sharp increase in the risk for noncardiac mortality with decreasing BMI values in the normal weight and underweight categories and a relatively flat association between BMI and the risk of noncardiac mortality for values in the overweight and obesity categories of the BMI scale. The association of BMI with cardiac mortality remained U-shaped but was attenuated after adjustment for epidemiological and clinical variables available. Conversely, the association of BMI with noncardiac mortality remained significant after adjustment in the multivariable risk model. This may suggest that important correlates that underlay the risk for noncardiac mortality may remain unaccounted for with the epidemiological and clinical information available in patients undergoing PCI. A previous study suggested that the improved survival after PCI in patients with moderately increased BMI was mostly due to a lower noncardiac mortality [31]. Third, the interaction testing showed a BMI-by-age interaction, suggesting an increased risk of all-cause and noncardiac mortality in patients >75 years of age and a lower BMI. This finding strongly suggests that being old and underweight signifies an increased risk of long-term mortality (particularly noncardiac mortality) after PCI. A previous study showed that lean patients had the highest short-term and long-term mortality after primary PCI across all age groups, whereas increased BMI was associated with higher mortality in patients <75 years of age [28]. The study suggested that BMI–mortality relationship should be age-contextualized. The interaction testing also showed a BMI-by-sex interaction suggesting a stronger association of lower BMI with noncardiac mortality in women compared with men. This may imply that women with a lower BMI may be at a particularly higher risk of long-term noncardiac mortality. Although the sex-related differences in the relationship between BMI and mortality remain poorly investigated, some studies have suggested a protective effect of moderate obesity after coronary intervention in men but not in women [29,32]. The finding that women with a lower weight (BMI < 25 kg/m^2^) had higher odds of dying from noncardiac causes than men may have implications for the explanation of sex-based differences in the outcomes of patients after PCI [33]. Although, the exact reasons for observing higher odds of dying from noncardiac causes in women with a lower weight remain unclear, frailty—a well-known risk factor for poor outcomes after PCI [34]—may be more prevalent among women with a lower weight. However, the hypothesis that frailty may confer a higher risk of noncardiac mortality particularly among women with a lower weight needs testing in future studies.

Limited and controversial evidence exists with respect to the association of BMI with long-term outcomes other than mortality after PCI [18,35,36,37]. A recent study showed that mortality and TVR were the main drivers of the increased risk of major adverse cardiac and cerebrovascular events observed in patients with a lower BMI after PCI [18]. Other studies did not find differences according to BMI in the risk for myocardial infarction, TLR or TVR after PCI [35,37]. There are also conflicting results with respect to the association of BMI with the risk for stent thrombosis after PCI [35,36,37,38]. Several studies did not find an increased risk for stent thrombosis according to BMI categories [35,37,38]. One study reported an inverse relationship between BMI and the risk for stent thrombosis after PCI [36]. Congruent with prior studies [35,37,38], our study did not find differences in the risk for myocardial infarction, TLR or TVR according to BMI categories up to 10 years of follow-up after PCI. However, we found a higher risk of definite stent thrombosis and NonTVR after PCI in overweight patients compared with normal weight patents even after adjusting for epidemiological and clinical variables. Although, the increased risk for stent thrombosis associated with a higher BMI needs further confirmation, a prothrombotic state promoted by poor response to antiplatelet drugs, aspirin [39] and/or clopidogrel [40] may offer some explanation. On the other hand, the increased risk of NonTVR in overweight patients may reflect the progression of atherosclerosis in non-treated coronary arteries, which may be promoted by overweight/obesity.

### Limitations of the Study

The study has several limitations. First, the current study represents a retrospective analysis even though the study patients were recruited in randomized prospective trials with strict criteria for inclusion, follow-up and event adjudication. Second, all analyses were based on a single assessment of BMI at the time of PCI. However, a recent study suggested that a single measurement of BMI was adequate and can satisfactorily predict prognosis after PCI [41]. Third, even though BMI is the most commonly used metric to assess obesity, it is considered a surrogate of body fat that is not specific for visceral fat. Some studies have suggested that a combination of BMI with waist circumference offers a better assessment of the long-term risk associated with obesity [42]. Fourth, patients were recruited for the study over a long time interval and they received a somewhat outdated therapy in terms of coronary stents and antithrombotic drugs. Moreover, conditional on the time of patients’ recruitment in the source studies, sodium-glucose cotransporter-2 inhibitors and glucagon-like peptide-1 receptor agonists were not prescribed at the time of patients’ recruitment and we have no information whether these drugs were prescribed during the follow-up. Thus, an eventual impact of these drugs on the BMI or prognosis after PCI cannot be addressed. Fifth, conditional on the inclusion criteria and the outcomes assessed in the source studies, outcomes like stroke or bleeding after PCI cannot be assessed in the setting of the current study. Finally, although we adjusted for epidemiological and clinical data available, residual confounding in the association of BMI with the outcomes cannot be refuted.

## 5. Conclusions

In conclusion, BMI was associated with 10-year mortality after PCI with a U-shaped relationship. The association of BMI with cardiac mortality was U-shaped but was attenuated after adjustment, whereas the association between BMI and noncardiac mortality was L-shaped and remained significant after adjustment for potential confounders. BMI in the overweight category was associated with an increased risk of definite stent thrombosis and NonTVR compared with patients in the normal weight category. There was a BMI-by-age interaction, suggesting a lower risk of all-cause and noncardiac mortality in patients >75 years of age and a BMI > 25 kg/m^2^ and a BMI-by-sex interaction suggesting a lower risk of noncardiac mortality in women with a BMI > 25 kg/m^2^.

## Figures and Tables

**Table 1:** Baseline characteristics.

Characteristic	Body Mass Index (kg/m^2^)				*p* Value
	<18.5 (n = 59)	18.5 to <25 (n = 1608)	25 to <30 (n = 2509)	≥30 (n = 1421)	
Body mass index (kg/m^2^)	17.7 [17.1–18.2]	23.4 [22.1–24.4]	27.3 [26.1–28.4]	32.2 [31.0–34.6]	<0.001
Age (years)	73.4 [62.5–80.1]	70.1 [62.8–78.0]	68.2 [60.8–74.8]	65.6 [57.4–72.7]	<0.001
Women	35 (59.3%)	503 (31.3%)	466 (18.6%)	325 (22.9%)	<0.001
History of arterial hypertension	40 (67.8%)	1054 (65.5%)	1688 (67.3%)	995 (70.0%)	0.073
History of hypercholesterolemia	32 (54.2%)	952 (59.2%)	1666 (66.4%)	967 (68.1%)	<0.001
Diabetes mellitus	9 (15.3%)	345 (21.5%)	687 (27.4%)	581 (40.9%)	<0.001
On insulin therapy	3 (5.08%)	112 (6.97%)	198 (7.89%)	222 (15.6%)	<0.001
On oral antidiabetic drugs	4 (6.78%)	176 (10.9%)	384 (15.3%)	261 (18.4%)	<0.001
Serum creatinine (mg/dl)	0.87 [0.78–1.00]	0.90 [0.80–1.10]	0.98 [0.80–1.10]	0.94 [0.80–1.11]	<0.001
Estimated GFR (ml/min/1.73 m^2^)	77.0 [58.3–91.6]	77.5 [59.8–90.1]	78.0 [61.6–91.6]	80.1 [62.1–93.2]	0.006
Current smoker	20 (33.9%)	278 (17.3%)	377 (15.0%)	262 (18.4%)	<0.001
Prior myocardial infarction	18 (30.5%)	446 (27.7%)	719 (28.7%)	447 (31.5%)	0.135
Prior coronary artery bypass surgery	4 (6.8%)	124 (7.7%)	267 (10.6%)	147 (10.3%)	0.011
Diagnosis at presentation					0.038
Chronic coronary disease	36 (61.0%)	903 (56.2%)	1517 (60.5%)	855 (60.2%)	
Acute coronary syndrome	23 (39.0%)	705 (43.8%)	992 (39.5%)	566 (39.8%)	
Number of coronary arteries narrowed					0.049
1	11 (18.6%)	263 (16.4%)	358 (14.3%)	208 (14.6%)	
2	21 (35.6%)	443 (27.5%)	684 (27.3%)	350 (24.6%)	
3	27 (45.8%)	902 (56.1%)	1467 (58.4%)	863 (60.8%)	
Left ventricular ejection fraction (%)	54.0 [40.5–60.0]	56.0 [46.0–62.0]	56.0 [48.0–62.0]	56.0 [46.0–61.0]	0.064

Data are median [25th; 75th percentiles] or number of patients (%). GFR = glomerular filtration rate.

**Table 2:** Ten-year clinical outcomes.

Events	Body Mass Index (kg/m^2^)				Hazard Ratio [95% Confidence Interval]		
	<18.5 (n = 59)	18.5 to <25 (n = 1608)	25 to <30 (n = 2509)	≥30 (n = 1421)	<18.5 vs. 18.5 to <25 kg/m^2^	25 to <30 vs. 18.5 to <25 kg/m^2^	>30 vs. 18.5 to <25 kg/m^2^
All-cause death	31 (59.7)	582 (39.1)	710 (31.1)	431 (33.8)	1.72 [1.20–2.47]	0.75 [0.67–0.83]	0.83 [0.73–0.93]
Cardiac death	14 (27.2)	355 (24.3)	441 (19.7)	266 (21.3)	1.15 [0.67–1.96]	0.78 [0.68–0.90]	0.86 [0.73–1.00]
Noncardiac death	17 (32.5)	227 (14.7)	269 (11.4)	165 (12.4)	2.33 [1.44–3.76]	0.75 [0.63–0.89]	0.82 [0.67–1.01]
Myocardial infarction	6 (10.3)	104 (6.7)	152 (6.3)	75 (5.5)	1.64 [0.72–3.73]	0.94 [0.73–1.21]	0.82 [0.61–1.10]
Definite stent thrombosis	0 (0.0)	7 (0.4)	28 (1.2)	18 (1.3)	-	2.59 [1.13–5.93]	2.97 [1.24–7.09]
Target lesion revascularization	6 (11.0)	287 (18.6)	449 (18.7)	261 (19.4)	0.56 [0.25–1.26]	1.01 [0.87–1.18]	1.05 [0.89–1.24]
Target vessel revascularization	7 (12.7)	326 (20.8)	591 (24.3)	340 (24.9)	0.58 [0.27–1.22]	1.19 [1.04–1.36]	1.22 [1.05–1.42]
Nontarget vessel revascularization	5 (9.2)	393 (25.2)	739 (30.6)	429 (31.6)	0.33 [0.14–0.80]	1.25 [1.10–1.41]	1.29 [1.12–1.48]

Data are the number of events with cumulative incidences calculated by the Kaplan–Meier method. For outcomes other than all-cause mortality, cumulative incidences were calculated after accounting for the competing risk of death. The normal weight group (BMI: 18.5 to <25 kg/m^2^) served as a reference.

**Table 3:** Results of multivariable Cox proportional hazards model applied for all-cause, cardiac and noncardiac mortality.

Characteristic	All-Cause Mortality		Cardiac Mortality		Noncardiac Mortality	
	HR [95% CI]	*p* Value	HR [95% CI]	*p* Value	HR [95% CI]	*p* Value
BMI category: underweight vs. normal weight	1.52 [1.06–2.20]	0.024	0.85 [0.46–1.57]	0.605	2.47 [1.50–4.05]	<0.001
BMI category: overweight vs. normal weight	0.82 [0.73–0.92]	<0.001	0.91 [0.79–1.05]	0.203	0.82 [0.68–0.99]	0.035
BMI category: Obesity vs. normal weight	0.96 [0.85–1.10]	0.580	1.00 [0.84–1.18]	0.989	0.99 [0.80–1.22]	0.918
Age (for 10-year increment)	1.92 [1.81–2.04]	<0.001	2.08 [1.92–2.25]	<0.001	1.71 [1.55–1.88]	<0.001
Women	0.87 [0.78–0.98]	0.017	0.96 [0.84–1.11]	0.613	0.73 [0.61–0.88]	0.001
Diabetes mellitus	1.50 [1.36–1.66]	<0.001	1.60 [1.41–1.82]	<0.001	1.35 [1.15–1.60]	<0.001
Arterial hypertension	0.92 [0.83–1.03]	0.134	0.95 [0.83–1.09]	0.487	0.88 [0.75–1.05]	0.153
Current smoking	1.54 [1.34–1.79]	<0.001	1.76 [1.46–2.11]	<0.001	1.24 [0.97–1.58]	0.090
Hypercholesterolemia	0.90 [0.81–0.99]	0.036	0.93 [0.82–1.06]	0.277	0.85 [0.72–0.99]	0.043
Previous myocardial infarction	1.11 [1.00–1.23]	0.065	1.07 [0.94–1.23]	0.312	1.17 [0.99–1.38]	0.065
Previous coronary artery bypass surgery	1.07 [0.92–1.23]	0.354	1.04 [0.86–1.25]	0.677	1.11 [0.89–1.40]	0.354
Presentation with acute coronary syndrome	0.99 [0.90–1.09]	0.606	1.01 [0.89–1.15]	0.844	0.96 [0.82–1.12]	0.606
Multivessel disease	1.24 [1.06–1.46]	0.014	1.22 [1.00–1.50]	0.046	1.25 [0.96–1.62]	0.091
Estimated GFR (for 30 mL/min/1.73 m^2^ decrement)	1.80 [1.67–1.94]	<0.001	1.78 [1.61–1.96]	<0.001	1.84 [1.64–2.07]	<0.001
Type of coronary vessel	1.00 [0.90–1.11]	0.675	0.97 [0.84–1.12]	0.667	1.04 [0.87–1.24]	0.675
ACC/AHA complex lesions	1.00 [0.90–1.12]	0.951	1.00 [0.87–1.15]	0.944	0.99 [0.84–1.18]	0.951
Left ventricular ejection fraction (10% decrement)	1.27 [1.22–1.32]	<0.001	1.33 [1.27–1.40]	<0.001	1.19 [1.12–1.27]	<0.001

ACC/AHA = American College of Cardiology/American Heart Association; CI = confidence interval; BMI = body mass index; GFR = glomerular filtration rate; HR = hazard ratio.

**Table 4:** Association of body mass index categories with clinical outcomes after adjustment in the Cox proportional hazards model.

Outcome	Body Mass Index Categories					
	Underweight vs. Normal Weight		Overweight vs. Normal Weight		Obesity vs. Normal Weight	
	HR [95% CI]	*p* Value	HR [95% CI]	*p* Value	HR [95% CI]	*p* Value
All-cause death	1.52 [1.06–2.20]	0.024	0.82 [0.73–0.92]	<0.001	0.96 [0.85–1.10]	0.580
Cardiac death	0.85 [0.46–1.57]	0.605	0.91 [0.79–1.05]	0.203	1.00 [0.84–1.18]	0.989
Noncardiac death	2.47 [1.50–4.05]	<0.001	0.82 [0.68–0.99]	0.035	0.99 [0.80–1.22]	0.918
Myocardial infarction	1.61 [0.67–3.87]	0.283	0.93 [0.72–1.20]	0.593	0.78 [0.57–1.06]	0.114
Definite stent thrombosis	-	-	2.43 [1.05–5.59]	0.038	2.21 [0.93–5.26]	0.073
Target lesion revascularization	0.90 [0.75–1.08]	0.253	0.90 [0.77–1.04]	0.152	0.90 [0.75–1.08]	0.253
Target vessel revascularization	0.60 [0.28–1.27]	0.184	1.09 [0.95–1.25]	0.239	1.08 [0.92–1.27]	0.357
Nontarget vessel revascularization	0.37 [0.15–0.89]	0.027	1.14 [1.01–1.29]	0.041	1.13 [0.97–1.31]	0.108

CI = confidence interval; HR = hazard ratio.

## Data Availability

The data that support the findings of this study are available from the corresponding author upon reasonable request.

## Supplementary Materials

The following supporting information can be downloaded at: https://www.mdpi.com/article/10.3390/jcm14051413/s1, Table S1. Procedural data. Table S2. Drug therapy at hospital discharge. Figure S1. Kaplan-Meier curves of cardiac and noncardiac mortality in patients groups according to categories of body mass index. Figure S2. All-cause mortality in subgroups according to age, sex, diabetes mellitus and clinical presentation. Figure S3. Noncardiac mortality in subgroups according to age, sex, diabetes mellitus and clinical presentation. Figure S4. Association between body mass index (BMI) with 10-year cardiac mortality in unadjusted (left panel) and adjusted (right panel) restricted cubic spline analysis. Figure S5. Association between body mass index (BMI) with 10-year noncardiac mortality in unadjusted (left panel) and adjusted (right panel) restricted cubic spline analysis.
