# Supplementary material for: Body Mass Index and 10-Year Clinical Outcomes After Percutaneous Coronary Intervention—Interaction with Age, Sex, Diabetic Status and Clinical Presentation
Source: J Clin Med. 2025 Feb 20;14(5):1413. doi: 10.3390/jcm14051413 (PMC11900202; doi:10.3390/jcm14051413)
Supplement: Supplementary file 1 [file jcm-14-01413-s001.zip › jcm-3430808-supplementary.pdf]

Supplemental Material

Table S1. Procedural data.

Table S2. Drug therapy at hospital discharge.

Figure S1. Kaplan-Meier curves of cardiac and noncardiac mortality in patients groups according to categories of body mass index.

Figure S2. All-cause mortality in subgroups according to age, sex, diabetes mellitus and clinical presentation.

Figure S3. Noncardiac mortality in subgroups according to age, sex, diabetes mellitus and clinical presentation.

Figure S4. Association between body mass index (BMI) with 10-year cardiac mortality in unadjusted (left panel) and adjusted (right panel) restricted cubic spline analysis.

Figure S5. Association between body mass index (BMI) with 10-year noncardiac mortality in unadjusted (left panel) and adjusted (right panel) restricted cubic spline analysis.

Table S1. Procedural data

Characteristic	Body mass index (kg/m^2)				P value
	<18.5 (n=79 lesions)	18.5 to <25 (n=2247 lesions)	25 to <30 (n=3483)	≥30 (n=1943 lesions)	
Vessel treated					0.005
LAD	31 (39.2%)	1050 (46.7%)	1533 (44.0%)	862 (44.4%	
LCX	15 (19.0%)	521 (23.2%)	949 (27.2%)	518 (26.7%)	
RCA	33 (41.8%)	676 (30.1%)	1001 (28.7%)	563 (29.0%)	
ACC/AHA complexity of lesions					0.759
A	5 (6.33%)	100 (4.45%)	144 (4.13%)	82 (4.22%)	
B1	16 (20.3%)	503 (22.4%)	786 (22.6%)	431 (22.2%)	
B2	44 (55.7%)	1158 (51.5%)	1775 (51.0%	965 (49.7%)	
C	14 (17.7%)	486 (21.6%)	778 (22.3%)	465 (23.9%)	
Bifurcational lesions	573 (25.5%)	951 (27.3%)	481 (24.8%)	19 (24.1%	0.170
Chronic occlusions	102 (4.54%)	194 (5.57%)	126 (6.48%)	3 (3.80%)	0.044
Reference diameter (mm)	2.80 [2.40-3.25]	2.76 [2.42-3.05]	2.77 [2.43-3.10]	2.85 [2.46-3.22]	<0.001
Pre-procedural minimal lumen diameter (mm)	0.98 [0.70-1.20]	0.92 [0.63-1.24]	0.91 [0.64-1.23]	0.92 [0.62-1.27]	0.811
Baseline stenosis (%)	64.4 [56.3-73.3]	64.6 [55.6-75.4]	65.3 [55.9-75.5]	66.0 [55.6-76.4]	0.327
Total stented length (mm)	23.0 [18.0-30.0]	24.0 [18.0-30.0]	24.0 [18.0-30.0]	24.0 [18.0-30.0]	0.891
Number of implanted stents	2.00 [1.00-2.00]	2.00 [1.00-2.00]	2.00 [1.00-2.00]	2.00 [1.00-2.00]	0.908
Residual stenosis (%)	10.4 [7.39-14.3]	10.9 [7.43-15.1]	10.9 [7.57-14.9]	11.0 [7.50-15.0]	0.955
Second generation DES	65 (82.3%)	1979 (88.1%)	3111 (89.3%)	1760 (90.6%)	0.013

Data are median with 25th-75th percentiles or counts (%); ACC=American College of Cardiology; AHA=American Heart Association; DES=drug-eluting stent; LAD=left anterior descending; LCX=left circumflex artery; RCA=right coronary artery

Table S2. Drug therapy at hospital discharge

Drug	Body mass index (kg/m^2)				P value
	<18.5 (n=59)	18.5 to <25 (n=1608)	25 to <30 (n=2509)	≥30 (n=1421)	
Aspirin	57/59 (98.3%)	1563/1597 (97.7%)	2444/2496 (98.1%)	1398/1412 (98.9%)	0.093
$P2Y_{12}$ inhibitors	54/58 (93.1%)	1496/1599 (93.6%)	2320/2487 (93.3%)	1316/1414 (93.1%)	0.071
Statins	56/58 (96.6%)	1508/1600 (94.2%)	2354/2490 (94.5%)	1343/1413 (95.0%)	0.721
Angiotensin-converting enzyme inhibitors	49/59 (83.1%)	1269/1608 (78.9%)	1920/2509 (76.5%)	1109/1421 (78.0%)	0.312
Angiotensin II receptor blockers (AT1)	4/58 (6.9%)	220/1594 (13.8%)	452/2485 (18.2%)	257/1413 (18.2%)	<0.001
Beta-blocking agents	55/58 (94.8%)	1494/1597 (93.6%)	2363/2491 (94.9%)	1344/1414 (95.0%)	0.231
Calcium channel blockers	6/58 (10.3%)	202/1596 (12.7%)	319/2484 (12.8%)	258/1413 (18.3%)	<0.001
Nitrates	0.0%	23/1593 (1.4%)	47/2483 (1.9%)	19 /1412(1.4%)	0.346
Diuretic drugs	39/58 (67.2%)	820/1592 (51.5%)	1240/2481 (50.0%)	833/1411 (59.0%)	<0.001
Insulin	3/58 (5.2%)	108/1592 (6.8%)	185/2483 (7.5%)	205/1411 (14.5%)	<0.001
Oral antidiabetic drugs	4/58 (6.9%	193/1594 (12.1%)	415/2484 (16.7%)	343/1411 (24.3%)	<0.001
Warfarin	5/59 (8.5%)	201/1597 (12.6%)	260/2494 (10.4%)	177/1412 (12.5%)	0.084

Data are numbers of patients (%)

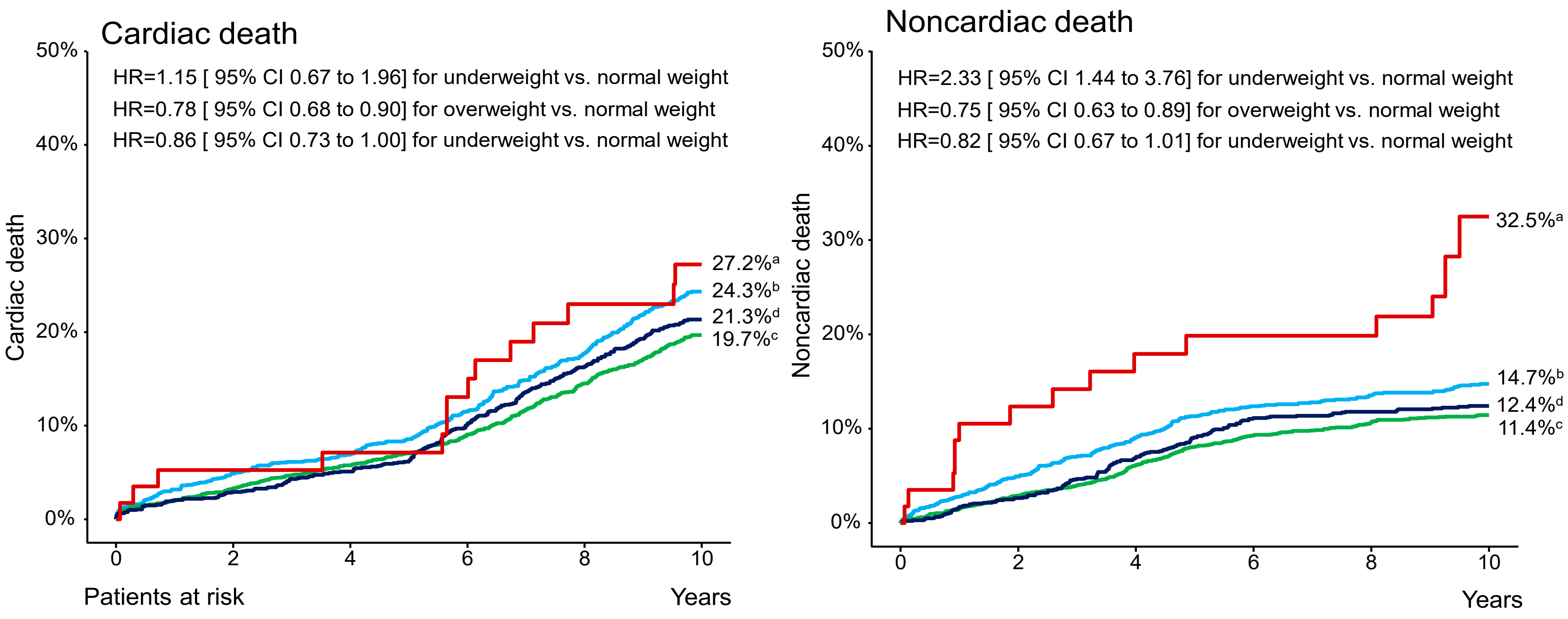

BMI category	0	2	4	6	8	10	0	2	4	6	8	10
Underweight[a]	59	45	40	34	28	17	59	45	40	34	28	17
Normal weight[b]	1608	1440	1309	1131	925	747	1608	1440	1309	1131	925	747
Overweight[c]	2509	2299	2111	1825	1565	1314	2509	2299	2111	1825	1565	1314
Obesity[d]	1421	1308	1186	969	829	689	1421	1308	1186	969	829	689

Figure S1. Kaplan-Meier curves of cardiac and noncardiac mortality in patient groups according to categories of body mass index (BMI)

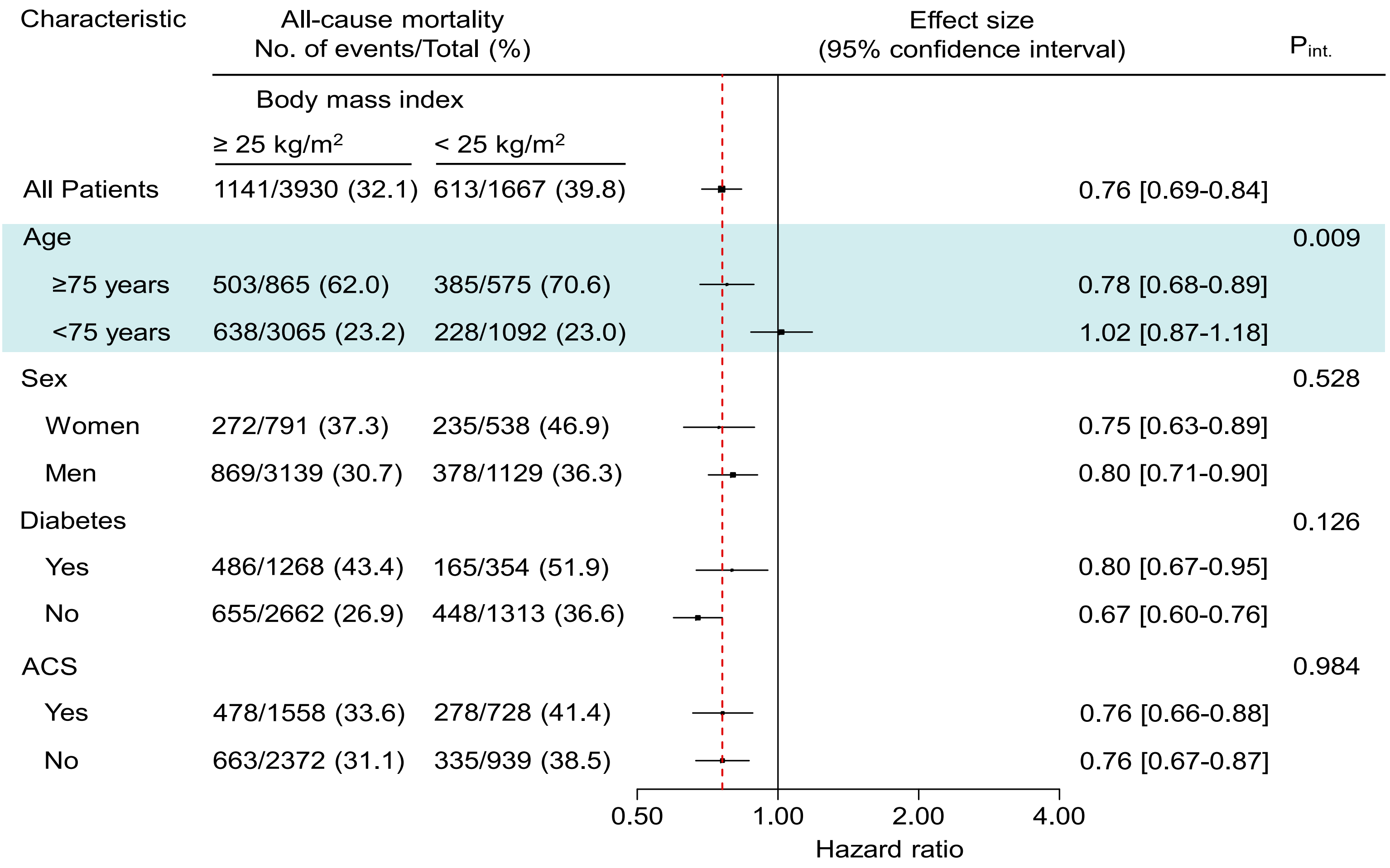

Figure S2. All-cause mortality in subgroups according to age, sex, diabetes mellitus and clinical presentation. ACS=acute coronary syndrome. The shaded area shows the significant interaction.

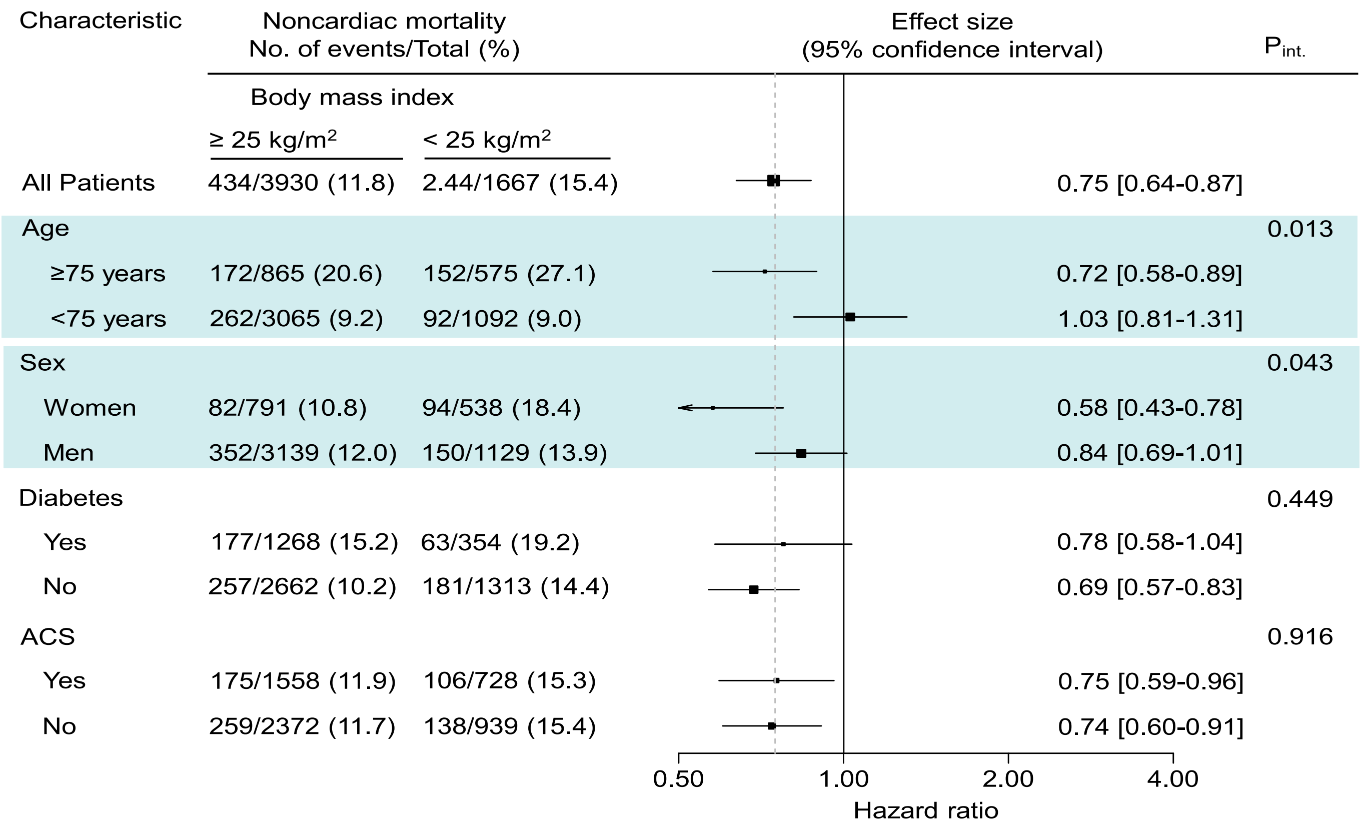

Figure S3. Noncardiac mortality in subgroups according to age, sex, diabetes mellitus and clinical presentation. ACS=acute coronary syndrome. The shaded areas show the significant interactions.

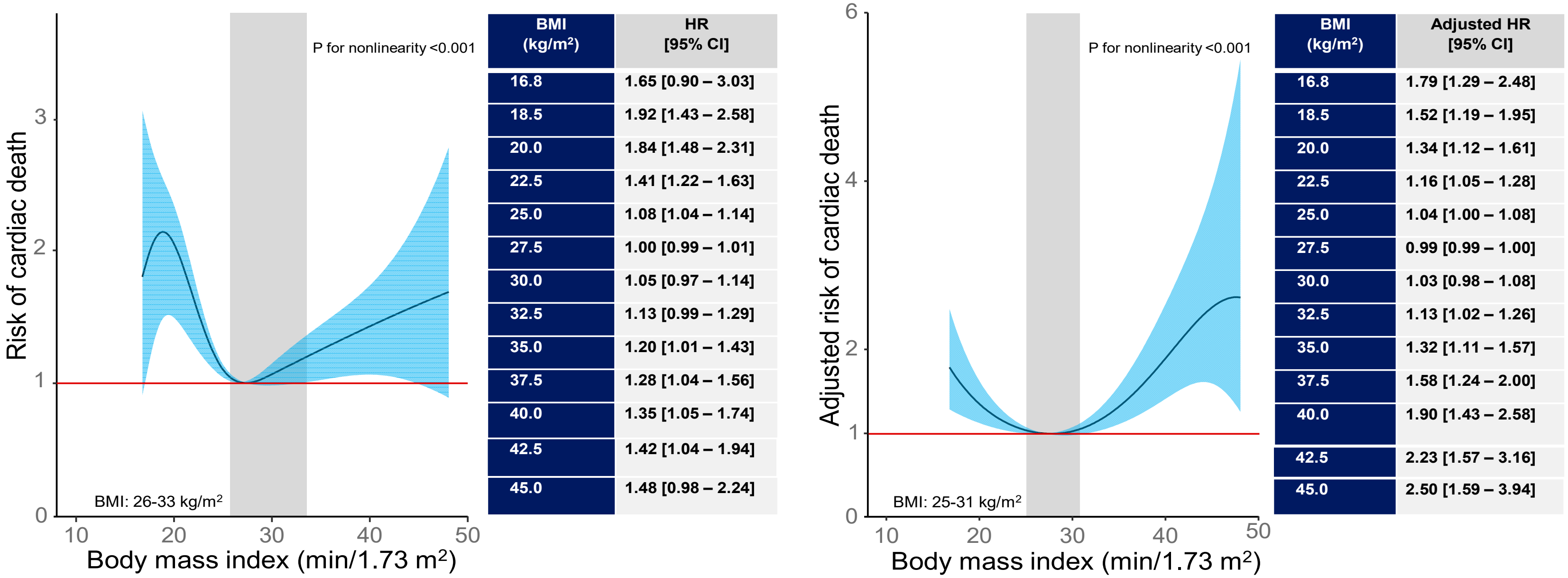

BMI (kg/m^2)	HR [95% CI]
16.8	1.65 [0.90 – 3.03]
18.5	1.92 [1.43 – 2.58]
20.0	1.84 [1.48 – 2.31]
22.5	1.41 [1.22 – 1.63]
25.0	1.08 [1.04 – 1.14]
27.5	1.00 [0.99 – 1.01]
30.0	1.05 [0.97 – 1.14]
32.5	1.13 [0.99 – 1.29]
35.0	1.20 [1.01 – 1.43]
37.5	1.28 [1.04 – 1.56]
40.0	1.35 [1.05 – 1.74]
42.5	1.42 [1.04 – 1.94]
45.0	1.48 [0.98 – 2.24]

BMI (kg/m^2)	Adjusted HR [95% CI]
16.8	1.79 [1.29 – 2.48]
18.5	1.52 [1.19 – 1.95]
20.0	1.34 [1.12 – 1.61]
22.5	1.16 [1.05 – 1.28]
25.0	1.04 [1.00 – 1.08]
27.5	0.99 [0.99 – 1.00]
30.0	1.03 [0.98 – 1.08]
32.5	1.13 [1.02 – 1.26]
35.0	1.32 [1.11 – 1.57]
37.5	1.58 [1.24 – 2.00]
40.0	1.90 [1.43 – 2.58]
42.5	2.23 [1.57 – 3.16]
45.0	2.50 [1.59 – 3.94]

Figure S4. Non-linear association between body mass index (BMI) with 10-year cardiac mortality in unadjusted (left panel) and adjusted (right panel) analysis. The spline curves show the risk of cardiac mortality across the whole spectrum of BMI values. The shaded areas show the BMI values associated with the lowest 10-year cardiac mortality. In unadjusted analysis, the BMI values lower than 26 kg/m^2 and higher than 33 kg/m^2 were associated with the increased risk of cardiac mortality. In adjusted analysis, the BMI values lower than 25 kg/m^2 and higher than 31 kg/m^2 were associated with the increased adjusted risk of cardiac mortality. The inserted tables on the right side of each graph show hazard ratios (HR) with 95% confidence interval (CI) for cardiac mortality in various BMI values.

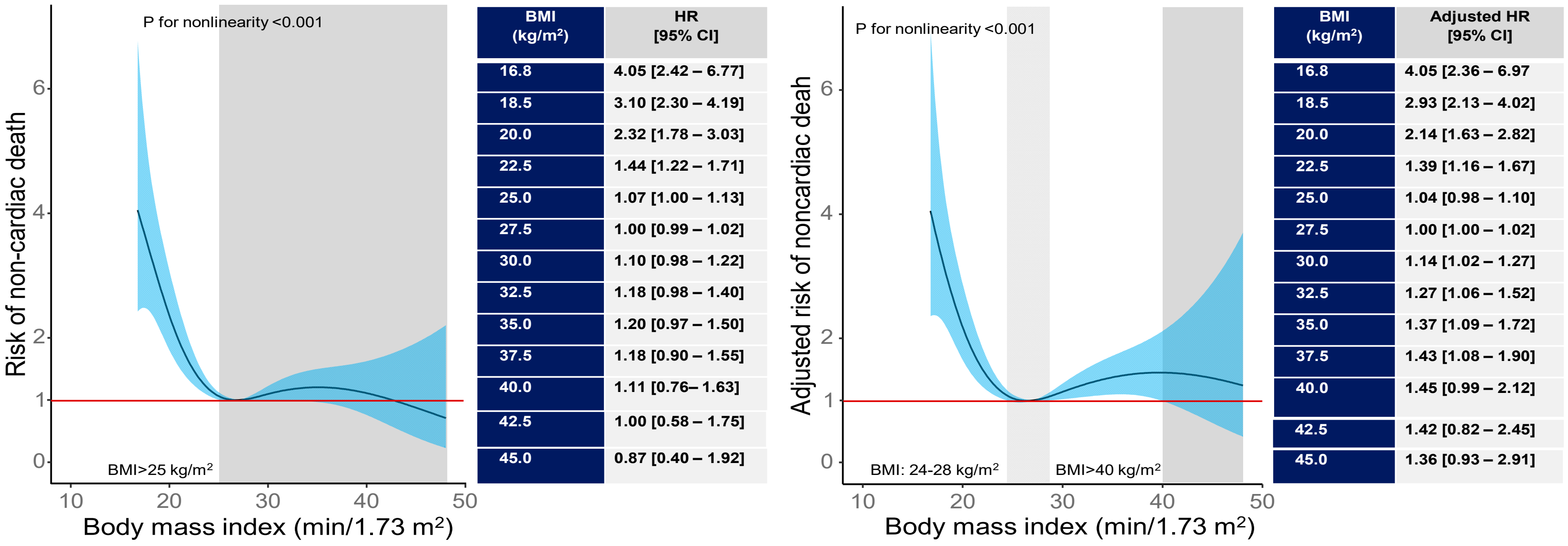

BMI (kg/m²)	HR [95% CI]
16.8	4.05 [2.42 – 6.77]
18.5	3.10 [2.30 – 4.19]
20.0	2.32 [1.78 – 3.03]
22.5	1.44 [1.22 – 1.71]
25.0	1.07 [1.00 – 1.13]
27.5	1.00 [0.99 – 1.02]
30.0	1.10 [0.98 – 1.22]
32.5	1.18 [0.98 – 1.40]
35.0	1.20 [0.97 – 1.50]
37.5	1.18 [0.90 – 1.55]
40.0	1.11 [0.76– 1.63]
42.5	1.00 [0.58 – 1.75]
45.0	0.87 [0.40 – 1.92]

BMI (kg/m²)	Adjusted HR [95% CI]
16.8	4.05 [2.36 – 6.97
18.5	2.93 [2.13 – 4.02]
20.0	2.14 [1.63 – 2.82]
22.5	1.39 [1.16 – 1.67]
25.0	1.04 [0.98 – 1.10]
27.5	1.00 [1.00 – 1.02]
30.0	1.14 [1.02 – 1.27]
32.5	1.27 [1.06 – 1.52]
35.0	1.37 [1.09 – 1.72]
37.5	1.43 [1.08 – 1.90]
40.0	1.45 [0.99 – 2.12]
42.5	1.42 [0.82 – 2.45]
45.0	1.36 [0.93 – 2.91]

Figure S5. Non-linear association between body mass index (BMI) with 10-year noncardiac mortality in unadjusted (left panel) and adjusted (right panel) analysis. The spline curves show the risk of noncardiac mortality across the whole spectrum of BMI values. The shaded areas show the BMI values not associated with the risk of noncardiac mortality. In unadjusted analysis, the BMI values higher than 25 kg/m^2 were not associated with increased risk of noncardiac mortality. In adjusted analysis, the BMI values between 24 to 28 kg/m^2 and higher than 40 kg/m^2 were not associated with the increased adjusted risk of noncardiac mortality. The inserted tables on the right side of each graph show hazard ratios (HR) with 95% confidence interval (CI) for noncardiac mortality in various BMI values.
